# A Text-Book of the Theory and Practice of Medicine

**Published:** 1894-06

**Authors:** 


*A Text-book of the Theory and Practice of Medicine.* By American Teachers. Edited by William Pepper, M.D. In Two Volumes. Vol. II. Pp. 1046. Philadelphia: W. B. Saunders. London: F. J. Rebman. 1894.

We are glad to welcome the second volume of a work "which represents truly the best teaching of the science and art of medicine at the present time," and to congratulate the publisher on the fact that he has been able to complete the text-book without delay. Having recently reviewed the first volume (March, 1894, p. 45), we need not describe the general appearance and purport of its twin brother. We have here 38 monographs by eight authorities, whose names are well known beyond the limits of their own country. It has been a real pleasure to read some of these condensed and complete accounts of what is known on the respective topics. It is difficult to criticise, and we do not wish to find fault without reason; but in future editions we should like to see further details on the treatment of phthisis, as we cannot accept the statement that climate and feeding are everything: antiseptics have now an immense weight of evidence in their favour, and cannot be dismissed with a passing and depreciatory reference. We also object to the following quotation, which expresses the writer's view on the treatment of hæmoptysis: "The insistence of absolute quiet demoralises the patients, keeping the patients on a low diet unnecessarily reduces their strength." We hold that both points are needful in combating the imminent peril of a large hæmoptysis, and we have no right to assume that "the bleeding will stop no matter what is done." Maintenance of quiet and physiological rest should be furthered by the use of morphia, the one drug needful.

A careful index of 45 pages considerably enhances the value of the work.

